# Skin expression of IL-23 drives the development of psoriasis and psoriatic arthritis in mice

**DOI:** 10.1038/s41598-020-65269-6

**Published:** 2020-05-19

**Authors:** Lili Chen, Madhura Deshpande, Marcos Grisotto, Paola Smaldini, Roberto Garcia, Zhengxiang He, Percio S. Gulko, Sergio A. Lira, Glaucia C. Furtado

**Affiliations:** 1 Precision Immunology Institute, Icahn School of Medicine at Mount Sinai, New York, NY USA; 2 Department of Pathology and Laboratory Medicine, Hospital for Special Surgery, New York, NY USA; 3 Division of Rheumatology, Department of Medicine, Icahn School of Medicine at Mount Sinai, New York, NY USA

**Keywords:** Inflammation, Immunopathogenesis

## Abstract

Psoriasis (PS) is a chronic skin inflammation. Up to 30% of the patients with PS develop psoriatic arthritis (PsA), a condition characterized by inflammatory arthritis that affects joints or entheses. Although there is mounting evidence for a critical role of interleukin-23 (IL-23) signaling in the pathogenesis of both PS and PsA, it remains unclear whether IL-23-induced skin inflammation drives joint disease. Here, we show that mice expressing increased levels of IL-23 in the skin (*K23* mice) develop a PS-like disease that is characterized by acanthosis, parakeratosis, hyperkeratosis, and inflammatory infiltrates in the dermis. Skin disease preceded development of PsA, including enthesitis, dactylitis, and bone destruction. The development of enthesitis and dactylitis was not due to high circulating levels of IL-23, as transgenic animals and controls had similar levels of this cytokine in circulation. IL-22, a downstream cytokine of IL-23, was highly increased in the serum of *K23* mice. Although IL-22 deficiency did not affect skin disease development, IL-22 deficiency aggravated the PsA-like disease in *K23* mice. Our results demonstrate a central role for skin expressed IL-23 in the initiation of PS and on pathogenic processes leading to PsA.

## Introduction

Psoriasis (PS), one of the most prevalent autoimmune diseases, is commonly associated with other conditions, including inflammatory bowel disease and arthritis^1,2^. Up to 30% of the patients with psoriasis have psoriatic arthritis (PsA), a potentially debilitating condition that causes joint damage and pain and have a significant impact in the quality of life^3^. PsA similarly affects both genders and in most populations typically occurs between ages 30 and 50^4^. PsA frequently affects joints of the hands, feet and knees causing joint swelling and pain often with diffuse involvement of the digits (dactylitis), and tendons/ligaments bone insertion sites (enthesis/enthesitis)^5^. A subset of patients develop axial disease with spondylitis and sacroiliitis^5^. Histologically, PsA is characterized by synovial hyperplasia and inflammation with cartilage and bone erosive changes.

The pathogenesis of PsA remains incompletely understood. In 70% of cases PS precedes the onset of PsA by several years^6^. There is evidence for a partial overlap between PS and PsA susceptibility genes, including HLA-Cw6 and several others identified in such as IL-13, NFKBIA and IL-23R^7–10^. Genome-wide association studies (GWAS) have also revealed genes uniquely associated with PsA such as IFNLR1^7–10^. The IL-23R gene has been one of the non-MHC genes most strongly and consistently associated with both PS and PsA^11^, and the IL-23/IL-17 pathway has been implicated in the development of aspects of the disease such as spondyloarthropathy^12^. IL-23 has been detected in the skin and joint synovial tissues of PsA patients^13^ and neutralization of IL-23 is effective in reducing PsA disease activity and severity^14^. IL-23 is a dimeric cytokine formed by two subunits, p19 and p40. IL-23 is produced by keratinocytes^15,16^ and activated antigen-presenting cells (APCs), including Langerhans cells, macrophages, and dendritic cells (DCs) that induce the differentiation of pathogenic Th17 cells^17^. Mice treated with IL-23 minicircle DNA in vivo developed arthritis^12,18^. Subsequently it was shown that high systemic levels of IL-23 could also lead to development of enthesitis and skin disease^12^. IL-23R^+^ T cells are found in the skin and in the entheses and have been suggested to react to systemic levels of IL-23 and play a pathogenic role in PsA^12^.

Identical T cell clones have been detected both in the skin and synovial tissues of PsA patients^19^ suggesting that a shared antigen might be driving immune responses in both sites. However, it remains unclear whether skin PS disease processes are required and drive the joint disease. In this report we show that mice expressing increased levels of IL-23 in the skin develop PS and PsA, including dactylitis, enthesitis and joint destruction. Of note, the skin abnormalities preceded development of enthesitis, dactylitis, and bone destruction. Surprisingly, the development of enthesitis and dactylitis was not predicated on circulating levels of IL-23, as transgenic animals and controls had similar levels of this cytokine in circulation. Together these results demonstrate an important role for IL-23 expressed in the skin in the initiation of the pathogenic processes leading to PsA.

## Results

### Generation of mice expressing IL-23 conditionally in keratinocytes

**Figure 1:**
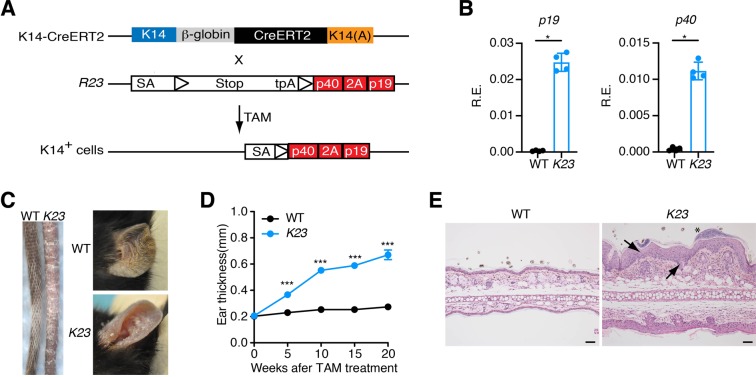
Transgenic expression of IL-23 by keratinocytes causes skin inflammation. (**A**) Scheme for generation of *K23* mice. *R23* mice were crossed with *K14*^*CreERT2*^ mice to generate *K23* mice. (**B**) Relative expression of *p19* and *p40* mRNA in the ears of WT and *K23* mice 8 weeks after TAM treatment (n = 4/group). *p < 0.05 by nonparametric Mann-Whitney test. (**C**) Representative macroscopic view of tails and ears of WT and *K23* mice after TAM treatment. *K23* mice treated with TAM showed inflammation in the tail and ears (n = 37 mice). (**D**) Quantification of the ear thickness after TAM treatment (n = 10–23 mice/time point). ***p < 0.001 compared with WT mice at the same time point by nonparametric Mann-Whitney test. (**E**) Representative H&E section of an ear of WT and *K23* mice 6 weeks after TAM treatment. Notice the presence of parakeratosis (arrow), acanthosis with regular elongation of rete ridges (arrow in the middle) and an abscess (asterisk) in the ear of *K23* mice treated with TAM. Scale bars, 50 μm.

To generate mice conditionally expressing IL-23 in the skin we first generated mice containing both *p40* and *p19* subunits of IL-23 (p40-2A-p19) in the ROSA26 locus downstream of a floxed STOP cassette (*R23* mice)^20^. *R23* mice were intercrossed with mice carrying a tamoxifen inducible Cre recombinase driven by the keratin-specific K14 promoter^21^ (K14^CreERT2^) to generate inducible *K23* mice (Fig. 1A). To promote cre-mediated excision of the STOP cassette and expression of IL-23 in skin cells, we treated *K23* mice with 3 cycles of tamoxifen in the food (TD.130968)^20^ (days 1–7, 14–21, and 28–35). To examine expression of the IL-23 transgene in the skin we extracted RNA from the ear of WT and *K23* mice after 8 weeks of TAM treatment and performed qPCR. As expected, we found that IL-23p40 and IL-23p19 expression was increased in the ears of mice that received TAM in *K23* mice compared to WT mice (Fig. 1B).

### Conditional expression of IL-23 in keratinocytes induces a psoriasis-like disease

The K14 promoter used to drive expression of the CreER gene is strongly active in dividing cells of epidermis, and targets the stem cell compartment^22^. Non-treated *K23* mice did not develop any phenotypes during 52 weeks of observation. However, PS-like skin phenotypes in the tails and ears were observed as early as 5 weeks after initiation of TAM treatment in *K23* mice, but not WT controls (Fig. 1C,D). These PS phenotypes included scaling and discoloration of the skin, ear swelling and patchy hair loss (Fig. 1C). Histological analyses of the skin of *K23* mice treated with TAM after 6 weeks, compared to WT mice, showed a psoriatic phenotype of acanthosis, parakeratosis, hyperkeratosis, and inflammatory infiltrates in the dermis (Fig. 1E), similar to human PS lesions^1^.

### Hallmarks of psoriasis are observed in the epidermis in *K23* mice

**Figure 2:**
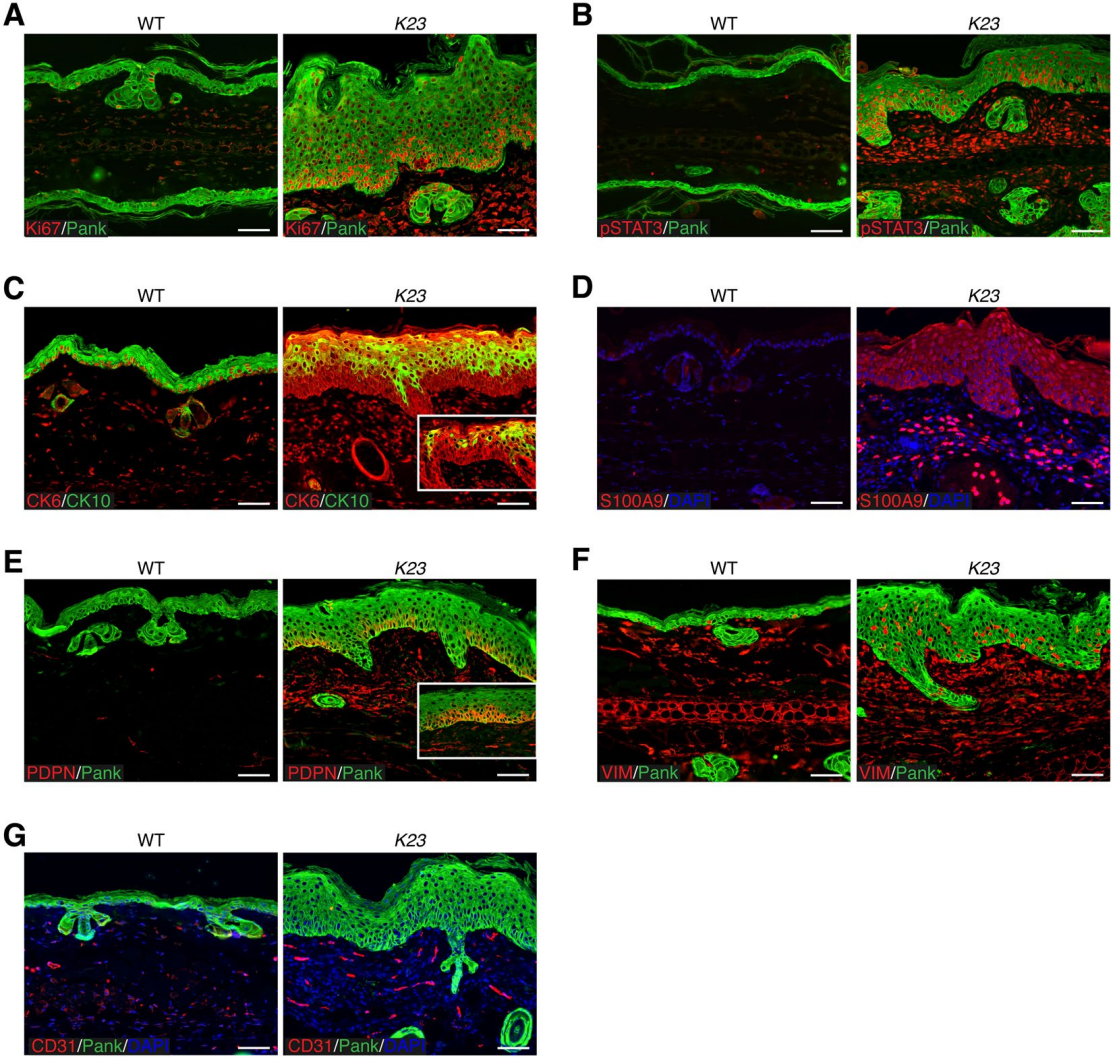
Hallmark features of PS reproduced in the skin of *K23* mice. (**A**,**B**) Increased expression of Ki67 (**A**) and pSTAT3 (**B**) in the ears of *K23* mice 6 weeks after TAM treatment. Pan-Keratin staining (PanK, green) showed increased epidermal keratinocytes in psoriatic lesions of *K23* mice. (**C**) Increased expression of CK6/CK10 in the ears of *K23* mice 6 weeks after TAM treatment. Notice areas showing co-expression CK6 and CK10 in the ear of *K23* mice (inset, arrowheads). (**D**–**F**) Immunofluorescence staining of ear of *K23* mice 20 weeks after TAM treatment. Increased expression of S100A9 (**D**), podoplanin (PDPN) (**E**), and vimentin (VIM) (**F**) in the epidermis of *K23* mice ears when compared to WT controls. (**G**) Increased umber of CD31^+^ vessels in the ear of *K23* mice when compared to controls. Scale bars, 50 μm.

Most clinical pathology in psoriasis is related to hyperproliferative and disturbed differentiation of epidermal cells. Similarly to what is observed in the skin of PS patients, the basal cell layer of the ear epidermis of *K23* mice was hyperproliferative, as indicated by Ki67 staining (Fig. 2A) and cells in the dermis and epidermis showed increased phosphorylation (activation) of STAT3 (pSTAT3) (Fig. 2B). Keratin 6 (CK6) is normally expressed by hyperproliferative epithelial cells^23^ whereas keratin 10 (CK10) is expressed by differentiated epithelial cells^24^. Similarly to what is observed in the human psoriatic lesions^25–27^, the ear skin of *K23* mice had cells co-expressing CK6 and CK10 (Fig. 2C). These results indicate that psoriatic skin is marked by hyperproliferation and aberrant differentiation of keratinocytes in *K23* mice after tamoxifen treatment.

To determine if *K23* developed a chronic inflammatory disease with similar characteristic to human psoriasis, we analyzed the expression of psoriasis markers in the ear skin of WT and *K23* mice 20 weeks after TAM treatment (Fig. 2D–G). The calcium-binding proteins S100A8 and S100A9 are highly expressed in PS skin^28–30^. Increased expression of S100A9 was also observed in the epithelial layer of the ear of *K23* mice when compared to WT littermate (Fig. 2D). Podoplanin, a well-known lymphatic endothelial marker, is also expressed in keratinocytes^31^. Similar to human PS lesions, the ears of *K23* mice with PS-like lesion had increased expression of podoplanin in the epidermis (Fig. 2E). As observed in human psoriatic lesions, vimentin^32^ expression was also increased in the epidermis of *K23* mice treated with TAM (Fig. 2F). Finally, psoriatic *K23* mice had increased CD31 expression in microvessels of papillary dermis suggesting increased angiogenesis as seen in PS skin (Fig. 2G). Together these results indicate that IL-23 expression in keratinocytes induces marked molecular expression changes in the skin that resemble those observed in human PS during the chronic phase.

### Characterization of immune infiltrates in the skin of *K23* animals

**Figure 3:**
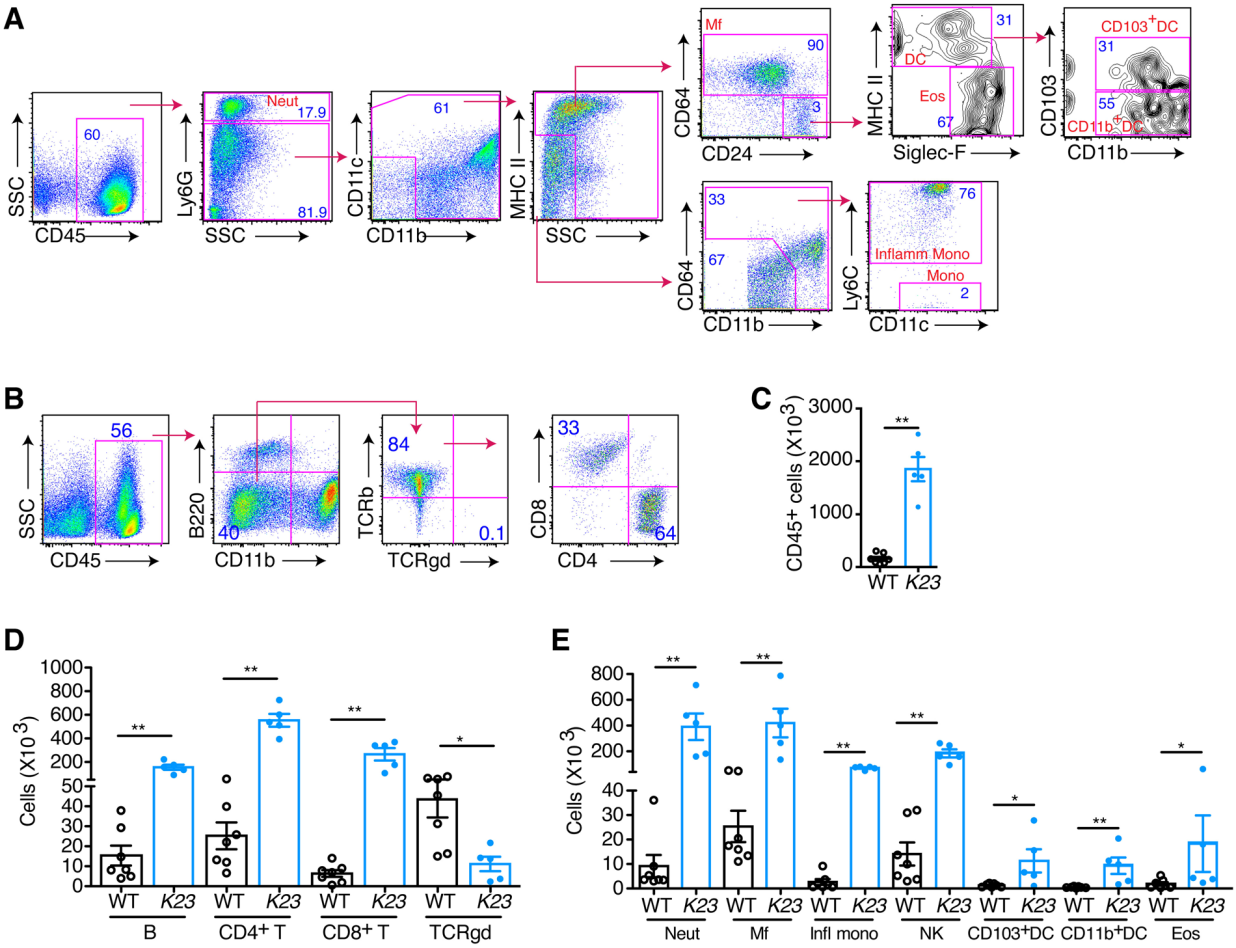
Multiple cell subsets are present in the inflamed skin. (**A**,**B)** Gating strategy for identification of myeloid (**A**) and lymphoid (**B**) subsets in the skin. (**C**–**E**) Total number of leukocytes (**C**), lymphoid (**D**) and other cells types (**E**) in the ear WT and *K23* mice 20 weeks after TAM treatment. Neutrophils (neut), macrophages (macr), inflammatory monocytes (infl mono), natural killer cells (NK), eosinophils (eos). n = 5–7 mice/group. Data are shown as mean± SEM, *p < 0.05, **p < 0.01 by nonparametric Mann-Whitney test.

To characterize the skin inflammatory infiltrates, we performed flow cytometric analysis using cells dissociated from the ears of *K23* and WT littermate mice 20 weeks after TAM treatment (Fig. 3). The flow cytometry gating strategy is shown in Fig. 3A,B. The ears of *K23* mice had 10 times more leukocytes than those of WT littermate controls (Fig. 3C). CD3^+^TCRαβ^+^ T cells (CD4^+^ T cells and CD8^+^ T cells) represented the major cell population that infiltrated the ears of *K23* mice (Fig. 3D). A significant decrease in the number TCRγδ T cells was observed in the skin of *K23* mice at this time-point (Fig. 3D). Neutrophils and macrophages were the major myeloid cell populations enriched in the skin of *K23* mice (Fig. 3E). A significant increase in the number of B cells (Fig. 3D), monocytes, DCs, NK, and eosinophils (Fig. 3E) was also observed in the psoriatic ears of *K23* mice. Together the results indicate that innate and adaptive immune cells accumulate in the ears of *K23* mice, similar to what is observed in human psoriatic lesions^33^.

### *K23* mice develop a PsA-like disease after PS

**Figure 4:**
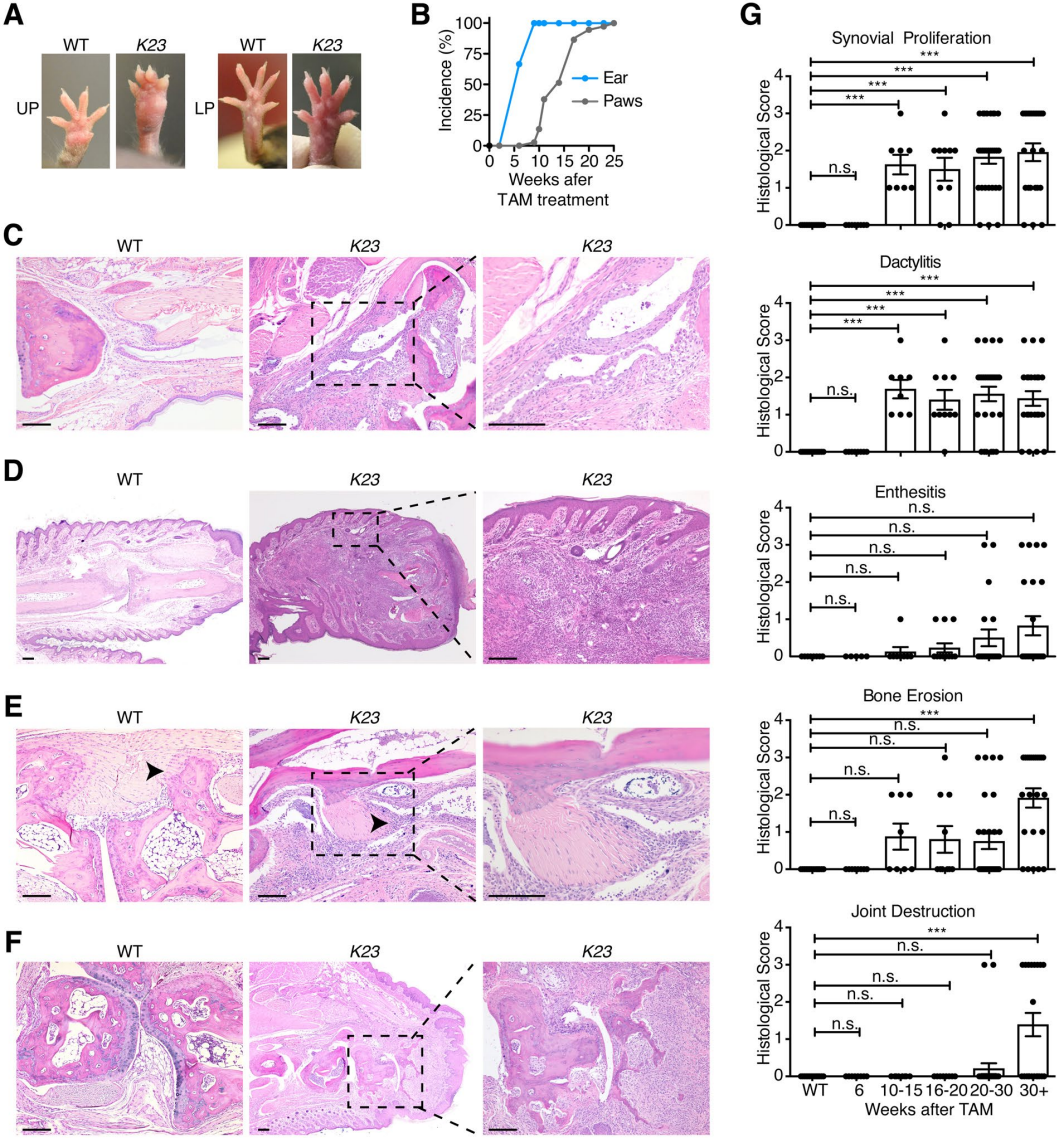
*K23* mice develop psoriatic-arthritis-like disease after TAM treatment. (**A**) Representative macroscopic view of paws of *K23* mice. *K23* mice treated with TAM develop inflammation in the upper (UP) and lower (LP) paws (n = 37 mice). (**B**) Incidence of disease in ears and paws of *K23* mice after TAM administration (n = 11–15 mice/group). (**C**–**F**) Representative H&E staining of paws and joints of WT and *K23* mice after TAM treatment. Arthritis in *K23* mice joints showed synovial proliferation (**C**), dactylitis (**D**), enthesitis (**E**), and joint destruction with acriisteiktsus (**F**). Scale bars, 100  μm. (**G**) Histopathological scores of WT and *K23* mice after TAM treatment at different time points. n = 4–16 mice/group. Data are shown as mean± SEM, ns, p > 0.05, ***p < 0.001 by one-way ANOVA followed by Dunnett’s multiple comparison test.

Up to 30% of the patients with psoriasis have psoriatic arthritis (PsA). As observed in patients with PsA, swelling of the digits (dactylitis) and arthritis with joint and paw swelling was also observed in 13% of *K23* mice after 10 weeks of TAM treatment reaching 100% by 25 weeks (Fig. 4A,B). These results indicate that *K23* mice develop arthritis after psoriasis (Fig. 4B). More importantly, the joints of *K23* mice treated with TAM had increased synovial hyperplasia (Fig. 4C), dactylitis (Fig. 4D), and enthesitis (Fig. 4E), when compared to WT mice. The pronounced inflammation of the joints of *K23* mice was associated with bone erosion and bone destruction including the typical distal phalanx acrosteolysis seen in PsA patients (Fig. 4F). Whereas psoriasis was observed in the skin as early as 6 weeks after TAM, synovial hyperplasia and dactylitis was observed 10 weeks after TAM treatment. Enthesitis and joint destruction were observed only after 19 weeks of TAM (Fig. 4G). These results indicate that psoriasis precedes the development of synovial inflammation and bone destruction in *K23* mice, as seen in most patients with PsA.

### Significant changes in circulating levels of cytokines during disease evolution

**Figure 5:**
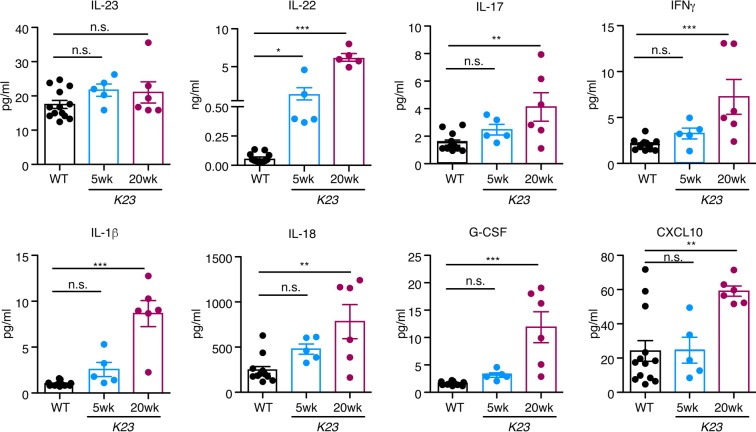
Peripheral cytokine expression after TAM treatment. WT and *K23* mice 5 weeks and 20 weeks after TAM treatment serum cytokine levels were analyzed by multiplex ELISA. n = 5–7 mice/group. Data are shown as mean± SEM, ns, p > 0.05, *p < 0.05, **p < 0.01, ***p < 0.001, by one-way ANOVA followed by Dunnett’s multiple comparison test.

**Figure 6:**
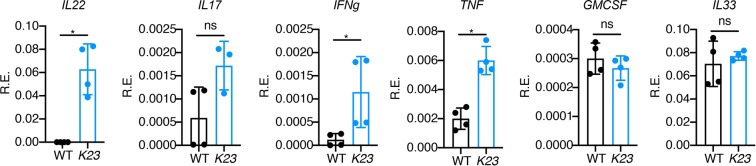
Relative expression of cytokines in the ears of K23 mice after TAM treatment. Relative expression of cytokines (IL-22, IL-17, IFNg, TNF, GMCSF and IL-33) in the ears of WT and K23 mice 8 weeks after TAM treatment (n = 3–4 mice/group). *p < 0.05, ns, not significant by non-parametric Mann-Whitney test.

It has been reported that high systemic levels of IL-23 induce psoriasis and spondyloarthritis in mice^12^. To examine if IL-23 could be detected in the serum at distinct phases of disease, we collected serum of *K23* mice at 5 and 20 weeks and used LUMINEX technology to quantify it. To our surprise, the circulating levels of IL-23 were not different between controls and *K23* mice (Fig. 5). We also investigated the serum levels of other inflammatory cytokines such as GCSF, GMCSF, CXCL1, CXCL2, CXCL10, IFNγ, IL-1α, IL-1β, IL-12p70, IL-15/IL-15R, IL-17, IL-18, IL-21, IL-22, IL-25, IL-27, Leptin, CCL2, CCL3, CCL4, CCL5, CCL7, MCSF, sRankl and TNF (Fig. 5). At 5 and 20 weeks the serum levels of IL-22, IL-17, IL-1β, IL-18 and GCSF but not the other cytokines, were significantly elevated. Levels of IFNγ and CXCL10 were increased only at 20 weeks. The most elevated cytokine in circulation was IL-22 (50 fold over control) (Fig. 5). In addition, we checked the expression of several cytokines (IL-22, IL-17, IFNg, TNF, GMCSF and IL-33) in the skin of *K23* mice 8 weeks after TAM treatment by qPCR analysis (Fig. 6). We found that the expression of the cytokines IL-22, IFNg and TNF were significantly increased in the skin of K23 mice compared to WT mice (Fig. 6). These results indicate that expression of IL-23 in the skin of *K23* mice does not significantly increase the levels of IL-23 in circulation, and suggest that the inflammatory process in the skin increases the circulating and local levels of several cytokines, most markedly IL-22.

### IL-22 is not required for development of the PsA-like phenotype

**Figure 7:**
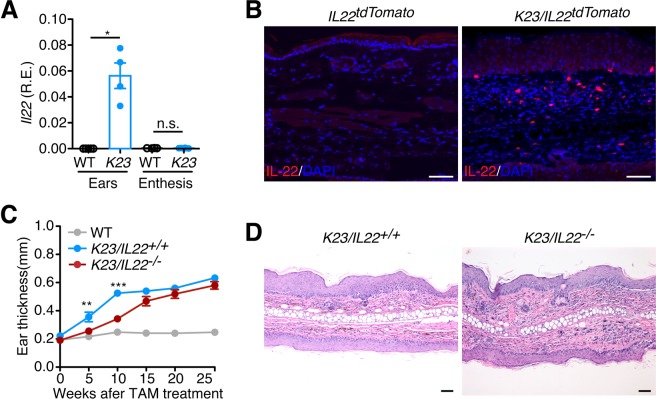
IL-22 is not required for development of skin inflammation. (**A**) Relative expression of IL-22 mRNA in the ears and enthesis of WT and *K23* mice 20 weeks after TAM treatment (n = 4-5 mice/group). Data are shown as mean± SEM, ns, p > 0.05, *p < 0.05, by nonparametric Mann-Whitney test. (**B**) Immunostaining of the ears of *IL22*^*tdTomato*^ and *K23/IL22* ^*tdTomato*^ mice 20 weeks after TAM treatment were stained with anti-tomato antibody. Cell nuclei were counterstained with DAPI (blue). Notice the accumulation of IL-22 positive cells in the ears of *K23* mice compared to WT mice. n = 5 per group. (**C**) Quantification of the ear thickness of WT, *K23/IL22*^*+/+*^, and *K23/IL22*^−/−^ mice after TAM treatment (n = 20–30 mice/time point). Data are shown as mean± SEM, **p < 0.01, ***p < 0.001 compared with WT mice at the same time point by nonparametric Mann-Whitney test. (**D**) Representative H&E sections of *K23/IL22*^*+/+*^ mice and *K23/IL22*^−/−^ mice ears 20 weeks after TAM treatment. Scale bars, 50 μm.

IL-22 is expressed in psoriatic skin lesions^34^ and within the inflamed diseased synovium^35^. To examine if IL-22 was expressed in the skin and joints of *K23* mice treated with TAM, we performed qPCR analysis. IL-22 was expressed in the ears but not in the enthesis of *K23* mice 20 weeks after TAM treatment (Fig. 7A). IL-22 is expressed by different hematopoietic and non-hematopoietic cell types, including αβ T cells, γδ T cells, NKT cells, and innate lymphoid cells (ILCs), macrophages, neutrophils, and fibroblasts^36^. To localize IL-22 expression in the skin, we intercrossed the *K23* mice with the *IL-22tdTomato* reporter mice^37^ (referred to as *K23/IL22tdTomato*). IL-22^+^ cells were detected in the cellular infiltrates in the dermis and occasionally in the epidermis of *K23/IL22tdTomato* but not in control *IL-22tdTomato* animals (Fig. 7B). IL-22 levels were elevated in circulation of *K23* mice suggesting that IL-22 could potentially have a role in pathogenesis. To define if expression of IL-22 was required for disease development, we generated *K23* mice deficient in IL-22 (referred to as *K23/IL-*22^−/−^ mice). After treatment with TAM, *K23/IL-*22^−/−^ mice were monitored for development of skin and joint phenotypes. *K23/IL-*22^−/−^ mice showed decreased ear thickness when compared to *K23/IL-*22^+/+^ mice 10 weeks after TAM, but this difference was not significant at later time points (Fig. 7C). Histological analysis of the skin of *K23/IL-*22^+/+^ and *K23/IL-*22^−/−^ 20 weeks after TAM treatment did not show differences, with mice showing similar levels of inflammatory infiltrates, acanthosis, parakeratosis, and hyperkeratosis (Fig. 7D). These results suggest that IL-22 is not required for development of skin inflammation.

### IL-22 deficiency aggravates the PsA-like disease in K23 mice

**Figure 8:**
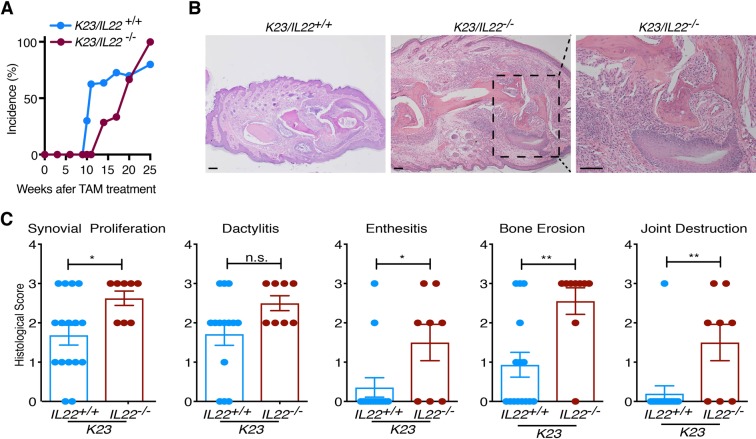
IL-22 deficiency aggravates the PsA-like disease in *K23* mice. (**A**) Incidence of disease in the paws of *K23/IL22*^*+/+*^ mice and *K23/IL22*^−/−^ mice after TAM treatment. (**B**) Lower paw histology of *K23/IL22*^*+/+*^ mice and *K23/IL22*^−/−^ mice 25 weeks after TAM treatment. Inset revealed erosive changes and destruction of the distal phalanx and adjacent joints (n = 8-9 mice/group). Scale bars, 100 μm. (**C**) Histological scores of *K23/IL22*^*+/+*^ mice and *K23/IL22*^−/−^ mice lower paws 25-30 weeks after TAM treatment. Data are shown as mean± SEM, ns, p > 0.05, * p < 0.05, **p < 0.01, by nonparametric Mann-Whitney test.

To study the role of IL-22 in the PsA-like disease development, we also analyzed development of PsA-like disease in *K23/IL-*22^−/−^ and *K23/IL-*22^+/+^ mice. There was no significantly difference in the incidence of development PsA-like disease in *K23/IL-*22^−/−^ and *K23/IL-*22^+/+^ mice (Fig. 8A). Histological analyses of the digits of *K23/IL-*22^−/−^ mice and *K23/IL-*22^+/+^ mice showed inflammation and bone erosion were present in both groups of mice 25 weeks after TAM treatment (Fig. 8B). However, histological scores focusing on PsA features such as synovial proliferation, enthesitis, bone erosion and joint destruction showed that disease was more severe and destructive in *K23/IL-*22^−/−^ when compared to *K23/IL-*22^+/+^ animals (Fig. 8C). Together, these results indicate that ablation of IL-22 was not protective, and appeared to cause a more severe PsA-like disease.

## Discussion

In this report we describe a novel mouse model of PS and PsA induced by selective and conditional expression of IL-23 in the skin (*K23* mice). Upon expression of IL-23, the animals developed a skin inflammatory disease that presented hallmarks of psoriasis such as acanthosis, parakeratosis, cellular infiltrates in the dermis and epidermis, hyperkeratosis and increased angiogenesis. Several weeks after the development of the skin PS phenotypes *K23* mice developed arthritis, enthesitis, and dactylitis. Similar findings occur in humans. Most patients with PsA develop joint disease after developing skin disease^6^.

In addition to the typical clinical and histologic findings of PS and PsA, the skin of *K23* mice expressed increased levels of established markers of PS skin disease, including increased levels of pSTAT3^38^, S100A8, S100A9^28–30^ and podoplanin^31^. Furthermore, *K23* mice developed the typical PsA dactylitis, arthritis, enthesis and acroosteolysis, which is a destruction of the distal phalanx almost only seen in PsA. *K23* mice also had increased expression of other key mediators of inflammation and joint destruction such as IL-1β, IL-6, IFNγ and CXCL10.

An association of IL-23 with skin inflammatory disease was first suggested by analysis of mice expressing IL-23-p19 from the CMV-Bactin promoter^39^. These animals presented a systemic inflammatory disease and died prematurely. The skin had a marked inflammatory infiltrate characterized by the presence of lymphocytes, macrophages, and neutrophils in the dermis and epidermis, with focal epithelial necrosis and ulceration. Subsequent experiments demonstrated that IL-23 injection *in situ*, or systemically via gene therapy, could also induce similar inflammatory phenotypes^12,40,41^. Together with results shown here, these results establish a role for IL-23 in the development of psoriasis, and suggest that IL-23 acts locally in the skin to do so.

The IL-23/IL-17 axis is currently considered to be crucial in the pathogenesis of psoriasis^42^. Human IL-23 is primarily produced by antigen-presenting cells and induces and maintains differentiation of Th17 cells and Th22 cells, a primary cellular source of proinflammatory cytokines such as IL-17 and IL-22, which mediate the epidermal hyperplasia, keratinocyte immune activation and tissue inflammation inherent in psoriasis^43^.

To further study the role of IL-23 in skin inflammation we engineered transgenic mice carrying the subunits of IL-23 (p19 and p40) preceded by a loxP-flanked STOP cassette in the ROSA26 locus (R23 mice). To promote expression of p19 and p40, we crossed these animals to mice expressing Cre recombinase in keratinocytes. All double positive mice died within the first few days of life^44^. To bypass this early lethality, we intercrossed the *R23* mice to mice expressing an inducible form of cre recombinase in keratinocytes, and treated the double positive mice, when adult, with TAM. All the mice survived treatment but developed skin inflammation and, unexpectedly, developed localized joint disease. All the hallmarks of the human condition are observed in the mouse model (skin involvement, enthesitis and bone changes) directly implicating IL-23 in the pathogenesis of PsA. The only notable difference between the mouse model and the human disease concerns the development of enthesitis. In human PsA enthesitis occurs prior to development of synovitis and/or bone changes, whereas in *K23* mice enthesitis is observed after synovial involvement. While the reasons for this differences are not clear at the moment our results clearly document that the skin disease precedes and drives the joint disease in mice, and as such, they have implications to the understanding of the human disease.

Development of spondyloarthropathy has been observed in animals that express high levels of IL-23 after hydrodynamic injection of IL-23^12^. IL-23R^+^CD3^+^CD4^−^CD8^−^Sca1^+^ cells were present in the enthesis and systemically elevated levels of IL-23 were associated with enthesitis and entheseal new bone growth, in the absence of synovitis^12^. Subsequent work confirmed the presence of IL-23R^+^ cells in the entheses^45^. These observations suggested that systemically elevated levels of IL-23 could promote enthesitis by acting on a IL-23r positive cell population present in the enthesis. In contrast with these observations, we show here that elevated systemic levels of IL-23 are not required to drive development of skin or joint disease in *K23* mice. IL-23 is readily detected in the skin of K23 mice, but blood levels of IL-23 were not different from those of control mice. IL-23 levels have been reported to be either increased^46^ or unchanged^47^ in patients with PsA, suggesting that, like in the *K23* mice, increased serum levels are not an absolute requirement for the development of PsA. We thus hypothesized that factors induced by IL-23 in the skin could be pathogenic and drive the joint disease. The main cytokines induced by IL-23 are IL-17 and IL-22.

IL-22 is induced by IL-23 and has been shown to have both pro- and anti-inflammatory properties. Injection of minicircles encoding IL-22 in mice can induce paw swelling suggesting a possible role of IL-22 in joint inflammation^12^. Additionally, IL-22 levels are significantly elevated in the synovial fluid of PsA patients compared to that of patients with osteoarthritis^35^. In animal models, IL-22 is required for the development of the Th17-mediated skin inflammation^48^, and it has been shown that transgenic mice expressing IL-22 develop a psoriasis-like skin phenotype^49^. Several studies have suggested that IL-22 can act in synergy with IL-17 or interferon γ (IFN γ) through an IL-23-dependent mechanism^40^ to amplify the inflammation observed in psoriatic skin^50^. The circulating levels of IL-22 were high in the *K23* mice treated with TAM, and thus it was reasonable to hypothesize that IL-22 could have a role in the *K23* mice joint inflammation. Our results, however, do not support the hypothesis that elevated levels of IL-22 are directly causative of joint inflammation as its deletion did not prevent paw swelling and bone destruction in *K23* mice. In fact, IL-22 deletion increased the severity of the joint disease and damage, suggesting that in this setting IL-22 may have a protective function. This is of great interest given that IL-22 has been reported to have anti-inflammatory properties in organ-specific rodent models of inflammation, such as pancreatitis and pneumonitis^36^. The role of IL-17 in this specific model remains to be examined, but it has been described that injection of minicircles encoding IL-17 into mice also does not promote joint disease^12^.

Two other models have been previously reported to induce a PS-like disease based on genetic manipulation, including skin overexpression of activated STAT3^51^ and skin double knockdown of JunB/c-Jun^52^. While both are helpful models to study disease, none clearly addressed the role of IL-23 in disease development. More recently, it was shown that the chronic skin inflammation observed in mice deficient in the neural Wiskott-Aldrich syndrome protein (N-WASP) is dependent on IL-23 producing keratinocytes^16^.

On the basis of the relationship between PS and the occurrence of PsA, it has been hypothesized that the skin immune mechanisms might contribute and ultimately trigger the onset of the articular manifestations^42^. Our present results support this hypothesis. We show that expression of IL-23 in the skin leads to development of skin disease that precedes development of arthritis. Whether this disease is driven by specific autoantigens shared between joint and skin as proposed^42^, or reflects increased generation of autoreactive T cells remains to be elucidated.

## Methods

### Ethics approval

All animal experiments in this study were approved by the Institutional Animal Care and Use Committee of Icahn School of Medicine at Mount Sinai, and were performed in accordance with the approved guidelines for animal experimentation at the Icahn School of Medicine at Mount Sinai (IACUC-2016-0509).

### Mice

*R23* mice^20^ and *IL-22*^−/−^ mice^44^ were described before. K14-creERT2 mice (CN 005107)^22^ were purchased from The Jackson Laboratory (Bar Harbor, ME). IL-22 tdTomato mice were provided by Dr. Scott Duram (NIH, Bethesda, MD). *R23* mice were crossed with K14-creERT2 mice to generate *K23* animals. Mice were maintained under specific pathogen-free conditions.

### Reverse-transcription polymerase chain reaction

Total RNA from tissues was extracted using the RNeasy mini Kit (Qiagen) according to the manufacturer’s instructions. Quantitative reverse-transcription polymerase chain reaction (RT-qPCR) experiments were performed as previously described^20,44,53^.

### Ear thickness

Ear thickness was measured at indicated time using digital calipers. An increase in ear thickness was used to indicate the extent of epidermal proliferation and inflammation.

### Histology and Immunostaining

Organs were dissected, fixed in 10% phosphate-buffered formalin, and processed for paraffin sections. Four-micrometer sections were stained with hematoxylin and eosin (H&E) for histology. Immunostaining studies were performed as described before^20,44,53^. Primary Abs used were anti-pan-Keratin, -Ki-67 (Abcam), -p-STAT3 (Cell Signaling), -cytokeratin 10, -cytokeratin 6 A (Biolegend), anti-CD45 (clone 30-F11), -F4/80 (clone BM8), -podoplanin (ebioscience), -tdTomato (LSBio). Secondary Abs used were Alexa Fluor 488 goat anti-mouse IgG1, Alexa Fluor 488 donkey anti-rat, Alexa Fluor 488 goat-anti rabbit, and Alexa Fluor 594 donkey anti-goat (Invitrogen).

### Flow cytometry

Ears were collected and processed as described^54^. All cells were first pre-incubated with anti-mouse CD16/CD32 for blockade of Fc γ receptors, then were washed and incubated for 40 min with the appropriate monoclonal antibody conjugates in a total volume of 200 μl PBS containing 2 mM EDTA and 2% (vol/vol) bovine serum. DAPI (Invitrogen) was used to distinguish live cells from dead cells during cell analysis. Stained cells were analyzed on a FACS Canto or LSRII machine using the Diva software (BD Bioscience). Data were analyzed with FlowJo software (TreeStar). The following fluorochrome-conjugated anti-mouse antibodies were used at indicated dilutions: CD103 (2E7, 1:200), CD11c (N418, 1:200), CD24 (30-F11, 1:200), CD11b (M1/70, 1:200), MHC-II (M5/114.15.2, 1:400), CD45 (30-F11, 1:200), CD64 (X54-5/7.1, 1:200), Ly6C (HK1.4, 1:200), TCRb (H57-597, 1:200), CD3 (145-2C11, 1:200), TCRgd (EBIOGL3, 1:200), B220 (RA3-6B2, 1:200), CD49b (DX5, 1:200) and FCeR1 (MAR-1, 1:200) were from eBioscience; Siglec-F (E50-2440, 1:200), Ly6G (1A8, 1:200), CD117 (2B8, 1:200), CD8 (53-6.7, 1:200), and CD4 (GK1.5, 1:200) were from BD Bioscience.

### Enzyme-linked immunosorbent assay

Chemokines and cytokines (GCSF, GMCSF, CXCL1, CXCL2, CXCL10, IFNγ, IL-1α, IL-1β, IL-12p70, IL-15/IL-15R, IL-17, IL-18, IL-21, IL-22, IL-23, IL-25, IL-27, Leptin, CCL2, CCL3, CCL4, CCL5, CCL7, MCSF, sRankl, and TNF) were measured in mouse plasma with ProcartaPlex Multiplex Immunoassays (eBioscience) according to the manufacturer’s protocol. Analysis was performed with the xMAP Technology by Luminex.

### Statistics

Differences between groups were analyzed with nonparametric Mann-Whitney test. For the comparison of more than two groups a one-way ANOVA followed by a Bonferroni multiple comparison test was performed. All statistical analyses were performed with GraphPad Prism 7 software.

## References

[1] Veale DJ, Fearon U (2018). The pathogenesis of psoriatic arthritis. Lancet.

[2] Fiorino G, Omodei PD (2015). Psoriasis and Inflammatory Bowel Disease: Two Sides of the Same Coin?. Journal of Crohn’s & colitis.

[3] Nestle FO, Kaplan DH, Barker J (2009). Psoriasis. The New England journal of medicine.

[4] Ritchlin CT, Colbert RA, Gladman DD (2017). Psoriatic Arthritis. The New England journal of medicine.

[5] Oliveira Mde F, Rocha Bde O, Duarte GV (2015). Psoriasis: classical and emerging comorbidities. Anais brasileiros de dermatologia.

[6] Gladman DD, Antoni C, Mease P, Clegg DO, Nash P (2005). Psoriatic arthritis: epidemiology, clinical features, course, and outcome. Annals of the rheumatic diseases.

[7] O’Rielly DD, Rahman P (2014). Genetics of psoriatic arthritis. Best practice & research. Clinical rheumatology.

[8] Stuart PE (2015). Genome-wide Association Analysis of Psoriatic Arthritis and Cutaneous Psoriasis Reveals Differences in Their Genetic Architecture. American journal of human genetics.

[9] Cargill M (2007). A large-scale genetic association study confirms IL12B and leads to the identification of IL23R as psoriasis-risk genes. American journal of human genetics.

[10] Liu Y (2008). A genome-wide association study of psoriasis and psoriatic arthritis identifies new disease loci. PLoS genetics.

[11] Nguyen, C. T., Bloch, Y., Skladanowska, K., Savvides, S. N. & Adamopoulos, I. E. Pathophysiology and inhibition of IL-23 signaling in psoriatic arthritis: A molecular insight. *Clinical immunology*, 10.1016/j.clim.2018.09.002 (2018). 10.1016/j.clim.2018.09.002 PMC6401348 30196070

[12] Sherlock JP (2012). IL-23 induces spondyloarthropathy by acting on ROR-gammat+ CD3+CD4-CD8- entheseal resident T cells. Nature medicine.

[13] Dolcino M (2015). Gene Expression Profiling in Peripheral Blood Cells and Synovial Membranes of Patients with Psoriatic Arthritis. PloS one.

[14] Kavanaugh A (2014). Ustekinumab, an anti-IL-12/23 p40 monoclonal antibody, inhibits radiographic progression in patients with active psoriatic arthritis: results of an integrated analysis of radiographic data from the phase 3, multicentre, randomised, double-blind, placebo-controlled PSUMMIT-1 and PSUMMIT-2 trials. Annals of the rheumatic diseases.

[15] Piskin G, Sylva-Steenland RM, Bos JD, Teunissen MB (2006). *In vitro* and *in situ* expression of IL-23 by keratinocytes in healthy skin and psoriasis lesions: enhanced expression in psoriatic skin. Journal of immunology.

[16] Li H (2018). Epigenetic control of IL-23 expression in keratinocytes is important for chronic skin inflammation. Nature communications.

[17] Gaffen SL, Jain R, Garg AV, Cua DJ (2014). The IL-23-IL-17 immune axis: from mechanisms to therapeutic testing. Nature reviews. Immunology.

[18] Adamopoulos IE (2011). IL-23 is critical for induction of arthritis, osteoclast formation, and maintenance of bone mass. Journal of immunology.

[19] Tassiulas I, Duncan SR, Centola M, Theofilopoulos AN, Boumpas DT (1999). Clonal characteristics of T cell infiltrates in skin and synovium of patients with psoriatic arthritis. Human immunology.

[20] Chen L (2018). Diet Modifies Colonic Microbiota and CD4(+) T-Cell Repertoire to Induce Flares of Colitis in Mice With Myeloid-Cell Expression of Interleukin 23. Gastroenterology.

[21] Wang X, Zinkel S, Polonsky K, Fuchs E (1997). Transgenic studies with a keratin promoter-driven growth hormone transgene: prospects for gene therapy. Proceedings of the National Academy of Sciences of the United States of America.

[22] Vasioukhin V, Degenstein L, Wise B, Fuchs E (1999). The magical touch: genome targeting in epidermal stem cells induced by tamoxifen application to mouse skin. Proceedings of the National Academy of Sciences of the United States of America.

[23] Weiss RA, Eichner R, Sun TT (1984). Monoclonal antibody analysis of keratin expression in epidermal diseases: a 48- and 56-kdalton keratin as molecular markers for hyperproliferative keratinocytes. The Journal of cell biology.

[24] Leigh IM, Purkis PE, Whitehead P, Lane EB (1993). Monospecific monoclonal antibodies to keratin 1 carboxy terminal (synthetic peptide) and to keratin 10 as markers of epidermal differentiation. The British journal of dermatology.

[25] Thewes M, Stadler R, Korge B, Mischke D (1991). Normal psoriatic epidermis expression of hyperproliferation-associated keratins. Archives of dermatological research.

[26] Mommers JM, van Rossum MM, van Erp PE, van De Kerkhof PC (2000). Changes in keratin 6 and keratin 10 (co-)expression in lesional and symptomless skin of spreading psoriasis. Dermatology.

[27] Korver JE, van Duijnhoven MW, Pasch MC, van Erp PE, van de Kerkhof PC (2006). Assessment of epidermal subpopulations and proliferation in healthy skin, symptomless and lesional skin of spreading psoriasis. The British journal of dermatology.

[28] Schonthaler HB (2013). S100A8-S100A9 protein complex mediates psoriasis by regulating the expression of complement factor C3. Immunity.

[29] Guttman-Yassky E (2008). Low expression of the IL-23/Th17 pathway in atopic dermatitis compared to psoriasis. Journal of immunology.

[30] Swamy M, Jamora C, Havran W, Hayday A (2010). Epithelial decision makers: in search of the ‘epimmunome’. Nature immunology.

[31] Honma M (2013). Podoplanin expression is inversely correlated with granular layer/filaggrin formation in psoriatic epidermis. The Journal of dermatology.

[32] Man, X. Y. *et al*. Analysis of epithelial-mesenchymal transition markers in psoriatic epidermal keratinocytes. *Open biology* **5**, 10.1098/rsob.150032 (2015). 10.1098/rsob.150032 PMC4554915 26269426

[33] Lowes MA, Suarez-Farinas M, Krueger JG (2014). Immunology of psoriasis. Annual review of immunology.

[34] Wolk K (2004). IL-22 increases the innate immunity of tissues. Immunity.

[35] Mitra A, Raychaudhuri SK, Raychaudhuri SP (2012). Functional role of IL-22 in psoriatic arthritis. Arthritis research & therapy.

[36] Dudakov JA, Hanash AM, van den Brink MR (2015). Interleukin-22: immunobiology and pathology. Annual review of immunology.

[37] Shen, W., Li, W., Hixon, J. A., Andrews, C. & Durum, S. K. Visualization of IL-22-expressing Lymphocytes Using Reporter Mice. *Journal of visualized experiments: JoVE*, 10.3791/54710 (2017). 10.3791/54710 PMC5352292 28190033

[38] Sano S, Chan KS, DiGiovanni J (2008). Impact of Stat3 activation upon skin biology: a dichotomy of its role between homeostasis and diseases. Journal of dermatological science.

[39] Wiekowski MT (2001). Ubiquitous transgenic expression of the IL-23 subunit p19 induces multiorgan inflammation, runting, infertility, and premature death. Journal of immunology.

[40] Zheng Y (2007). Interleukin-22, a T(H)17 cytokine, mediates IL-23-induced dermal inflammation and acanthosis. Nature.

[41] Chan JR (2006). IL-23 stimulates epidermal hyperplasia via TNF and IL-20R2-dependent mechanisms with implications for psoriasis pathogenesis. The Journal of experimental medicine.

[42] Boutet, M. A., Nerviani, A., Gallo Afflitto, G. & Pitzalis, C. Role of the IL-23/IL-17 Axis in Psoriasis and Psoriatic Arthritis: The Clinical Importance of Its Divergence in Skin and Joints. *International journal of molecular sciences* **19**, 10.3390/ijms19020530 (2018). 10.3390/ijms19020530 PMC5855752 29425183

[43] Puig L (2017). The role of IL 23 in the treatment of psoriasis. Expert review of clinical immunology.

[44] Chen L (2019). Interleukin 22 disrupts pancreatic function in newborn mice expressing IL-23. Nature communications.

[45] Reinhardt A (2016). Interleukin-23-Dependent gamma/delta T Cells Produce Interleukin-17 and Accumulate in the Enthesis, Aortic Valve, and Ciliary Body in Mice. Arthritis & rheumatology.

[46] Celis R (2012). Synovial cytokine expression in psoriatic arthritis and associations with lymphoid neogenesis and clinical features. Arthritis research & therapy.

[47] Przepiera-Bedzak H, Fischer K, Brzosko M (2015). Serum IL-6 and IL-23 Levels and Their Correlation with Angiogenic Cytokines and Disease Activity in Ankylosing Spondylitis, Psoriatic Arthritis, and SAPHO Syndrome. Mediators of inflammation.

[48] Ma HL (2008). IL-22 is required for Th17 cell-mediated pathology in a mouse model of psoriasis-like skin inflammation. The Journal of clinical investigation.

[49] Wolk K (2009). IL-22 and IL-20 are key mediators of the epidermal alterations in psoriasis while IL-17 and IFN-gamma are not. Journal of molecular medicine.

[50] Rutz S, Eidenschenk C, Ouyang W (2013). IL-22, not simply a Th17 cytokine. Immunological reviews.

[51] Sano S (2005). Stat3 links activated keratinocytes and immunocytes required for development of psoriasis in a novel transgenic mouse model. Nature medicine.

[52] Zenz R (2005). Psoriasis-like skin disease and arthritis caused by inducible epidermal deletion of Jun proteins. Nature.

[53] He Z, Chen L, Furtado GC, Lira SA (2018). Interleukin 33 regulates gene expression in intestinal epithelial cells independently of its nuclear localization. Cytokine.

[54] Jensen KK (2005). The human herpes virus 8-encoded chemokine receptor is required for angioproliferation in a murine model of Kaposi’s sarcoma. Journal of immunology.

